# Evaluation of VCAM-1 Targeted Naringenin/Indocyanine Green-Loaded Lipid Nanoemulsions as Theranostic Nanoplatforms in Inflammation

**DOI:** 10.3390/pharmaceutics12111066

**Published:** 2020-11-09

**Authors:** Elena Valeria Fuior, Cristina Ana Mocanu, Mariana Deleanu, Geanina Voicu, Maria Anghelache, Daniela Rebleanu, Maya Simionescu, Manuela Calin

**Affiliations:** 1 “Medical and Pharmaceutical Bionanotechnologies” Laboratory, Institute of Cellular Biology and Pathology “Nicolae Simionescu” of the Romanian Academy, 050568 Bucharest, Romania; elena.fuior@icbp.ro (E.V.F.); cristina.mocanu@icbp.ro (C.A.M.); geanina.voicu@icbp.ro (G.V.); maria.anghelache@icbp.ro (M.A.); daniela.rebleanu@icbp.ro (D.R.); maya.simionescu@icbp.ro (M.S.); 2 “Liquid and Gas Chromatography” Laboratory, Department of Lipidomics, Institute of Cellular Biology and Pathology “Nicolae Simionescu” of the Romanian Academy, 050568 Bucharest, Romania; mariana.deleanu@icbp.ro; 3 Faculty of Biotechnologies, University of Agronomic Sciences and Veterinary Medicine of Bucharest (UASVM), 050568 Bucharest, Romania

**Keywords:** lipid nanoemulsions, naringenin, vascular cell adhesion molecule (VCAM)-1, inflammation, targeted delivery, lipopolysaccharides, nanoparticles, Indocyanine Green

## Abstract

Naringenin, an anti-inflammatory citrus flavonoid, is restrained from large-scale use by its reduced water solubility and bioavailability. To overcome these limitations, naringenin was loaded into lipid nanoemulsions directed towards vascular cell adhesion molecule (VCAM)-1, exposed by activated endothelium, and delivered intravenously in a murine model of lipopolysaccharide (LPS)-induced inflammation. To follow the in vivo bio-distribution, naringenin-loaded nanoemulsions were labeled with near-infrared probe Indocyanine Green (ICG). Based on ICG fluorescence, a VCAM-1-dependent retention of nanoemulsions was detected in the heart and aorta, while ultra-high-performance liquid chromatography (UHPLC) measurements showed a target-selective accumulation of naringenin in the heart and lungs. Correlated, fluorescence and UHPLC data indicated a mixed behavior of the VCAM-1 directed nanoparticles, which were driven not only by the targeting moiety but also by passive retention. The treatment with naringenin-loaded nanoemulsions reduced the mRNA levels of some inflammatory mediators in organs harvested from mice with acute inflammation, indicative of their anti-inflammatory potential. The data support a novel theranostic nanoplatform for inflammation, the naringenin/ICG-loaded nanoparticles that either by passive accumulation or effective targeting of the activated endothelium can be employed for imaging inflamed vascular areas and efficient delivery of the encapsulated therapeutic agent.

## 1. Introduction

Naringenin, a citrus flavonoid with therapeutic potential in various inflammation-related pathologies [1], is restrained from large-scale use by its poor water solubility and bioavailability [2]. To overcome these limitations, an emerging trend is to encapsulate naringenin (Nar) into diverse inclusion complexes and/or nanoformulations in order to be administered by various routes. For example, complexation with β-cyclodextrin (β-CD) or its derivatives increased the solubility and stability of naringenin [3,4]. The complexed flavonoid (Nar/ β-CD) reportedly exerts a potent anti-inflammatory action in various experimental models [5,6,7]. Thus, it suppresses TNF-α production, both in vitro in LPS-stimulated RAW 264.7 macrophages and in vivo in a murine abdominal writhes test [5]. It also inhibits p75 Neurotrophin Receptor (NTR) and c-jun N-terminal kinase (JNK) pathways in a mouse model of nerve regeneration [6]. Nar/ β-CD reduces the expression of vascular endothelial growth factor (VEGF), cyclooxygenase (COX)-2, phosphoinositide 3-kinase (PI3K), p38 mitogen-activated protein kinase (MAPK), matrix metalloproteinase (MMP)-2, and MMP-9 in choroidal neovascularization in rats [7]. It is noteworthy that, in all these cases, at the same concentration, complexed naringenin was more effective than the free compound. Different strategies were employed to incorporate naringenin in nanoparticles designed for applications that span a large therapeutic spectrum, including antidiabetic [8], antitumoral [9], neuroprotective [10,11], and UV protective [12]. In addition, besides the oral delivery [8,9,13], alternative routes such as nasal [10,11] or topical [12,14] are considered. As recently reviewed, the main strategies to deliver nanoparticles (NP) incorporating naringenin are based on: polyvinylpyrrolidone (PVP), chitosan, poly(ε-caprolactone) (PCL), hyaluronic acid, and solid lipid NP [15]. Anti-inflammatory effects were reported for PVP-coated naringenin-loaded NP in LPS-stimulated RAW 264.7 macrophages, through the downregulated expression of Nuclear Factor κB (NF-κB) via p38MAPK signaling pathway [16]. When administered intravenously (i.v.) in male Sprague Dawley rats, naringenin loaded in PVP-NP inhibited inducible NO synthase (iNOS) and COX-2 and reduced the levels of inflammatory cytokines tumor necrosis factor (TNF)-α, interleukin (IL)-6, IL-1beta, and monocyte chemoattractant protein (MCP)-1 [17].

In line with these directions of investigation, in a previous study, we designed lipid nanoemulsions (LN) loaded with naringenin, targeted towards the activated endothelium by attaching a recognition peptide for the inducible vascular cell adhesion molecule-1 (VCAM-1) [18]. We chose LN as a delivery nanosystem, as its hydrophobic core would be more suitable to accommodate higher amounts of the nonpolar molecule of naringenin than a nanoliposome that ensures a hydrophilic milieu, surrounded by the lipid bilayer, and more appropriate for water-soluble drugs. Therein, we reported that VCAM-1 targeted nanoemulsions (V-Nar/LN) further decreased the activation of TNF-α exposed human endothelial cells as compared with their non-targeted (Nar/LN) counterparts. Moreover, we found that flavonoid-loaded LN are functional, i.e., they reduce the monocyte infiltration through activated human endothelial cells by a mechanism involving the decrease in the nuclear translocation of NF-κB and the decline in the production of MCP-1 chemokine. Further, these naringenin-loaded nanoparticles did not affect cell viability and displayed hemocompatibility on erythrocytes from two animal models: C57BL/6 and ApoE-deficient mice [18].

Based on these in vitro results, herein we further questioned whether these naringenin-loaded nanoformulations have the capacity to reduce inflammation in vivo upon intravenous (i.v.) administration in a murine model of LPS-induced inflammation. To this aim, we examined (i) whether following i.v. injection, the VCAM-1 targeted naringenin-loaded nanoparticles localize preferentially at sites of the inflamed endothelium, and (ii) if naringenin incorporated into nanoemulsions retains its anti-inflammatory potential.

Due to the lack of spectral properties of naringenin, we envisioned a strategy to trace the localization of the nanoemulsions based on the near infrared (NIR) probe Indocyanine Green (ICG). ICG, an FDA approved chemical for assessing cardiac and hepatic function, is currently investigated as a promising tool in developing biomedical applications in imaging and theranostics [19,20]. In recent years, ICG was intensely studied in relationship with the diagnosis and therapy of atherosclerosis [21,22], as well as of various tumors using targeted and photoactivatable nanoparticles [23,24].

We report here the successful co-incorporation of naringenin and ICG into lipid nanoemulsions and the selective accumulation of naringenin delivered by VCAM-1 targeted nanoparticles (V-Nar/ICG/LN) to endothelium-rich organs, the heart and the lungs of the mice with LPS-induced inflammation. Naringenin-loaded nanoemulsions have a therapeutic potential as revealed by the significant reduction in the gene expression of tumor necrosis factor alpha (TNF)-α, interleukin (IL)-1β, monocyte chemoattractant protein (MCP)-1, Regulated upon Activation, Normal T Cell Expressed and Presumably Secreted (RANTES), and VCAM-1 in organs harvested from mice with acute LPS-induced inflammation. Based on their characteristics, V-Nar/ICG/LN could be employed as a theranostic nanoplatform in inflammation-related diseases.

## 2. Materials and Methods

### 2.1. Reagents and Consumables

The main reagents and consumables used in this study were provided by commercial suppliers, as specified: naringenin (catalog no. N5893, purity > 95%), hesperetin (catalog no. W431300, purity > 95%), soybean oil, sodium 4-[2-[(1E,3E,5E,7Z)-7-[1,1-dimethyl-3-(4-sulfonatobutyl) benzo[e]indol-2-ylidene]hepta-1,3,5-trienyl]-1,1-dimethyl benzo[e]indol-3-ium-3-yl]butane-1-sulfonate (Indocyanine Green), lipopolysaccharide (LPS, serotype E coli O111:B4), SYBR-Green were from SIGMA-Aldrich (St Louis, MO, USA); soy phosphatidyl choline (SPC), 1,2-distearoyl-sn-glycero-3-phosphoethanolamine-N-[Maleimide(PolyethyleneGlycol) 2000](Ammonium salt) (Mal-PEG-DSPE) and 1,2-dipalmitoyl-sn-glycero-3-phosphoethanolamine-N-(lissamine rhodamine B sulfonyl) (ammonium salt) (Rhodamine-PE) from Avanti Polar Lipids (Alabaster, AL, USA); VCAM-1 recognition peptide with sequence VHPKQHRGGSKGCC from GeneCust (Dudelange, Luxembourg); fetal bovine serum (FBS) from Gibco (ThermoFisher Scientific); glycerin from Carl Roth (GmbH, Germany); Tris (2-carboxyethyl) phosphine (TCEP) from ThermoFisher Scientific (Waltham, MA, USA); SpectraPor dialysis membrane (cut-off 500-1000 Da) was from Spectrum Labs (Spectrum Europe BV, Breda, Netherlands); 100 kDa cutoff Amicon centrifugal filter columns from Millipore (Billerica, MA, USA). Trizol was from Ambion; primers were from Eurogentec (Liège, Belgium); M-MLV Reverse Transcriptase and Taq DNA Polymerase from Invitrogen (ThermoFisher Scientific, Waltham, MA, USA). Deionized water (18.2 MΩ/cm) was prepared in house using Milli-Q system from Millipore (Watford, UK). HPLC grade solvents were from Merck (Kenilworth, NJ, USA).

### 2.2. Preparation of Lipid Nanoemulsions

#### 2.2.1. Preparation of Naringenin-Loaded Lipid Nanoemulsions

**Figure 1:**
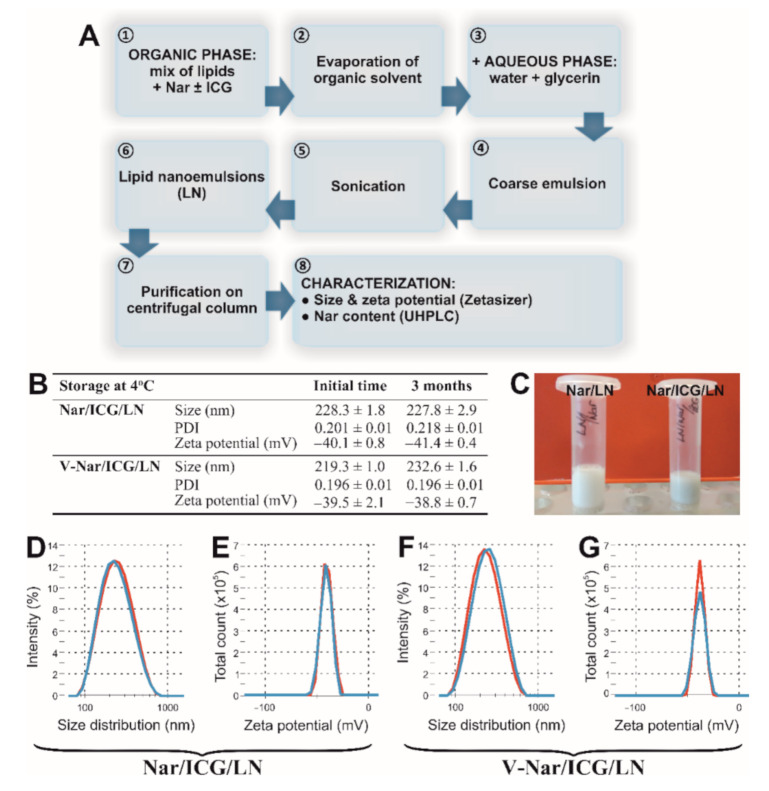
(**A**) Flow chart for the preparation and characterization of non-targeted naringenin-loaded lipid nanoemulsions (LN), either incorporating ICG (for biodistribution studies) or not (for evaluating the anti-inflammatory effect in a mouse model). (**B**) Physico-chemical characterization of lipid nanoemulsions incorporating naringenin and ICG. Summary of sizes, PDIs and zeta potentials for non-targeted (Nar/ ICG/LN) and VCAM-1 targeted (V-Nar/ICG/LN) nanoemulsions at the initial time of preparation and after 3-month storage at 4 °C. Standard deviations correspond to independent measurements for at least *n* = 6. (**C**) Photograph depicting Nar/ICG/LN (right) as compared with Nar/LN (left). (**D**,**F**) Particle size intensity distribution of non-targeted Nar/ICG/LN (**D**) and targeted V-Nar/ICG/LN (F) nanoemulsions as measured by dynamic light scattering. (**E**,**G**) Zeta potential distribution of non-targeted (**E**) and targeted (**G**) nanoemulsions measured by electrophoretic light scattering. Red curves correspond to the initial time, and blue curves represent measurements after 3-month storage at 4 °C.

Non-targeted naringenin-loaded lipid nanoemulsions (Nar/LN) were prepared through a procedure standardized in our laboratory to generate reproducible batches in terms of dimensions, zeta potential, and encapsulation efficiency, using the ultrasonication method as recently described [18]. For this, the organic phase, containing lipids dissolved in chloroform and a fixed volume of ethanol containing naringenin, was evaporated in vacuum on a rotary evaporator (Laborota 4000, Heidolph) at 40 °C. The residual was reconstituted in the aqueous phase, containing water and glycerin, following by sonication for 10 min using a UPH200H probe-type sonicator (Hielscher). The final formula of nanoemulsions contained 9.8 mM SPC, 0.2 mM Mal-PEG-DSPE and 0.5% *v/v* soybean oil, and 10% glycerin. The flow chart depicting the preparation procedure is shown in Figure 1A.

For the fluorescent labeling of the lipid nanoemulsions, 1.5 mol % Rhodamine-PE was added after LN preparation from an ethanol stock solution and incubated for 30 min at room temperature in the dark.

#### 2.2.2. Preparation of VCAM-1 Targeted Naringenin-Loaded Nanoemulsions

The preparation of VCAM-1 targeted naringenin-loaded nanoemulsions was previously described [18]. Briefly, VCAM-1 recognition peptide [25] was coupled to the maleimide group at the distal end of Mal-PEG-DSPE on the surface of non-targeted nanoparticles by incubating the nanoemulsions with the TCEP-reduced peptide. The coupling reaction (1 µg peptide: 1 µmole total lipid) was performed overnight at 4 °C in coupling buffer (20 mM sodium phosphate, pH 6.7, 30 mM NaCl, and 2 mM EDTA). Subsequently, the blockage of unreacted sites was achieved by the addition of a 100-fold molar excess of L-cysteine for 30 min at room temperature. The residual free L-cysteine was removed by centrifugation using 100 kDa cut-off Amicon filters.

#### 2.2.3. Preparation of Naringenin/ICG-Loaded Nanoemulsions

Naringenin-loaded nanoemulsions containing ICG (Nar/ICG/LN) were prepared by the same ultrasonication method as Nar/LN, adding ICG (150 µM) together with naringenin in the initial organic mix (Figure 1A). Non-incorporated naringenin and ICG were removed with the aid of 100 kDa cut-off Amicon centrifugal filters.

### 2.3. Characterization of Lipid Nanoemulsions

#### 2.3.1. Size and Zeta Potential

The size and zeta potential of lipid nanoemulsions were measured using a ZetaSizer NanoZS instrument (Malvern Instruments, Malvern, UK) equipped with a 633 nm laser.

The size of nanoparticles was evaluated by dynamic light scattering. Measurements were performed at 10 µM total lipid concentration in water at 25 °C, under automatic attenuator selection, with the following values for the physico-chemical parameters: viscosity of dispersant (water) 0.8872 cP, water dielectric constant 78.5, refractive indices: 1.45 for phospholipids and 1.33 for water. For each sample, reported size-averages of intensity distributions represent means of three runs, each of these based on 15 measurements.

For zeta potential measurements, a Universal Dip Cell (ZEN1002) was immersed into the sample after measuring the size. The zeta potential was calculated using the Smoluchowski model based on measurements of electrophoretic light scattering under automatic voltage selection. The results are presented as the averages of three recorded measurements, together with the standard deviation. Each individual record represents the average of 15 measurements. The results were analyzed using the build-in Zetasizer Software 7.12 (Malvern Instruments).

As a proof for the stability of Nar/ICG/LN and V-Nar/ICG/LN, their dimensions and zeta potentials after 3-month storage at 4 °C were checked.

#### 2.3.2. Amount of VCAM-1 Targeted Peptide Coupled to the Surface of Lipid Nanoemulsions

The concentration of coupled peptide was quantitated as previously described [26]. The amount of VCAM-1 recognizing peptide attached to the surface of Nar/LN was measured indirectly by detecting the amount of peptide that remained uncoupled after purification on the Amicon Ultra 100 KDa column. UHPLC measurements were done with an Agilent Technologies UHPLC 1290 Infinity instrument equipped with a binary pump, autosampler, column oven, and diode array UV/VIS detector. Separation was performed on an Eclipse Plus ZORBAX C18 column narrow bore RR (150 × 2.1 mm, 3.5 µm) with the column oven temperature kept at 25 °C. The mobile phase consisted of 0.1% trifluoroacetic acid in water (solvent A) and 0.1% trifluoroacetic acid in acetonitrile (solvent B) with a gradient elution of 5% B (0–0.8 min), 5–26% B (0.8–8 min), 26% B (8–10 min), and 26–5% B (10–12 min), at a flow rate of 0.25 mL/min. The detection was performed at the wavelength 220 nm. System control and data analysis were carried out using Agilent ChemStation software (B.04.02 Version, Agilent Technologies, Santa Clara, CA, USA).

#### 2.3.3. Naringenin Content in Lipid Nanoemulsions

The encapsulation efficiency (EE) of naringenin into LN was calculated based on the formula EE (%) = (Total naringenin − Free naringenin)/Total naringenin. Total concentration corresponded to the content in lipid nanoemulsions before separation by centrifugation using Amicon column, while the free naringenin was retrieved in the filtrate post-centrifugation.

Naringenin concentration was determined on a UHPLC (Agilent Technologies 1290 Infinity). Volumes of 5 μL were injected on an Eclipse Plus ZORBAX C18 column (150 × 2.1 mm, 3.5 µm). The mobile phase consisted of solvent A (water) and solvent B (acetonitrile). The gradient conditions were as follows: 0–1 min (23% B); 1–9 min (23–60% B); 9–10 min (60% B); 10–12 min (60–23% B); 12–14 min (23% B), at a flow rate of 0.25 mL/min. The temperature of the column was controlled at 30 °C and the detection wavelength was set at 290 nm. Data acquisition was carried out using Agilent ChemStation software (B.04.02 Version, Agilent Technologies, Santa Clara, CA, USA).

### 2.4. Biodistribution of Naringenin/ICG-Loaded Nanoemulsions

#### 2.4.1. Animal Model

Male C57BL/6 mice (Charles River Laboratories, USA), 12-week old with 20–25 g weight, were housed in the specific-pathogen-free (SPF) animal facility of the Institute, maintained at 24 °C constant ambient temperature, with 12-h alternating cycles dark/light. Animals were provided standard chow and water ad libitum. To induce the systemic inflammation, the mice were given LPS (0.5 mg/kg body weight), in a total volume of 150 µL phosphate buffer saline (PBS) by retro-orbital injection.

The experiments were approved by the Ethics Committee of the Institute of Cell Biology and Pathology “Nicolae Simionescu” and received the authorization no. 497/2020 from the National Sanitary Veterinary and Food Safety Authority. The studies were conducted according to the UE Directives, annex to Directive 86/69, Appendix A of European Convention for the protection of vertebrate animals and for experimental and other nature purposes (European Treaty Series (ETS) No. 123), Strasbourg, 2006.

#### 2.4.2. Localization of Naringenin/Icg-Loaded Nanoemulsions by Ex Vivo Imaging

A total of four hours after the LPS injection, the mice received a second retro-orbital injection, in the other eye, of ICG-labeled non-targeted (Nar/ICG/LN) or targeted (V-Nar/ICG/LN) naringenin-loaded LN containing 6 mg naringenin/75 µmoles lipid/862.5 µg ICG/kg body weight or an equivalent dose of free ICG. Mice receiving only sterile PBS or only LPS were used as controls for fluorescence background. One hour after nanoparticles administration, the animals were anesthetized using ketamine/xylazine and exsanguinated through open heart puncture. Perfusion with ice-cold PBS in the left ventricle was performed to remove the systemic blood. The brain, heart, aorta, aortic root, lung, spleen, liver, and kidneys were harvested and the ICG fluorescence was analyzed with the imaging system IVIS Spectrum Caliper 200. For spectral unmixing option, the following excitation/emission filter pairs: 710 nm/800 nm, 710 nm/820 nm, 710 nm/840 nm, 745 nm/800 nm, 745 nm/820 nm, and 745 nm/840 nm were used for ICG.

Quantification of the fluorescent radiant efficiency [(radiance of the fluorescent emission per incident excitation intensity (p/s/cm^2^/sr)/(μW/cm^2^)] was performed with the region of interest (ROI) tool of Living Image 4.3.1 software. Afterward, the organs were weighed, and frozen in liquid nitrogen until further processing for the quantification of naringenin content.

#### 2.4.3. Measurement of Naringenin Content in Organs

Tissue homogenates were obtained utilizing a Silent Crusher M homogenizer (Heidolph). Quantitation of naringenin was performed using the UHPLC method adapted from [27] with some modifications using hesperetin as the internal standard. The sample was prepared as follows: in 1.5 mL Eppendorf tube were added: 100 µL internal standard, 100 µL naringenin standard or 100 µL tissue homogenate/plasma, 100 µL MilliQ water, and 200 µL 0.5% formic acid. The mix was vigorously vortexed for 30 s and was loaded into preconditioned SampliQ C18 ODS 100 mg cartridges. After passing through with a flow rate of 1–2 drops/second, the cartridge was washed with 1 mL water at the same flow rate. Elution into a clean tube was achieved with 1 mL methanol, and the solvent was evaporated under nitrogen flow. The residue was reconstituted in 100 µL mix acetonitrile: water (80:20 *v/v*), vortexed for 1 min and centrifuged at 13,000 ×*g* for 10 min at room temperature. Naringenin concentration was measured on a UHPLC Agilent Technologies 1290 Infinity instrument equipped with diode-array detector (DAD) and 4 channel binary pump. Chromatographic separation was undertaken on a ZORBAX Eclipse Plus C18 column narrow bore RR (150 × 2.1 mm, 3.5 µm) at 30 °C and a mobile phase rate of 0.25 mL/minute. The mobile phase consisted of water (A) and acetonitrile (B), and elution was performed with the following step gradient: 0–1 min, 23% B; 1–9 min, 23–60% B; 9–10 min, 60% B; 10–12 min, 60–23% B; 12–14 min, 23% B. Injected sample volume was 10 µL, and detection wavelength was 290 nm. Agilent ChemStation software was used for data acquisition and processing. To quantitate naringenin content of the samples, the same protocol was used, except the sample was added into the tube instead of naringenin standard.

### 2.5. Assessment of the Anti-Inflammatory Effects of Naringenin-Loaded Nanoemulsions

#### Quantitative RT-PCR

Mice received a single retro-orbital injection with LPS (0.5 mg/kg body weight) and non-targeted (Nar/LN) or VCAM-1 targeted (V-Nar/LN) naringenin-loaded LN containing 6 mg naringenin/75 µmoles lipid/kg body weight in a volume of 125 µL PBS. As controls, mice receiving VCAM-1 targeted plain nanoemulsions (V-LN) (75 µmoles lipid/kg body weight), sterile PBS or LPS were used. At 24 h after treatment, the mice were anesthetized, exsanguinated, and sacrificed. The organs (brain, heart with aorta, lung, spleen, liver, and kidneys) were harvested and frozen in liquid nitrogen until further investigations. Total RNA extraction was achieved using Trizol reagent, and reverse transcription of mRNA was performed with the High Capacity cDNA Reverse Transcription Kit (ThermoFisher Scientific, Waltham, Massachusetts, USA). Amplification of cDNA, equivalent to 50 ng total RNA, was performed in RT-PCR reactions on a ViiA7 instrument (Applied Biosystems), with the final concentrations of the reagents in a 10 µL volume: 1.5 mM MgCl_2_, 1 µM of each primer, 0.25 mM dNTP, 0.5 U Taq DNA Polymerase (Invitrogen). Experiments were performed in 96-well plates, using SYBR Green (1×) as a fluorescent probe. The 2^−ΔΔCT^ method was used to quantify fold changes in the expression of the investigated genes [28]. ACTB (β-actin) was used as a reference gene. The detailed sequences of the primers for analyzing the murine genes of interest are reported in Table 1.

#### 2.6. Statistical Analysis

Results were expressed as means ± standard deviation. Statistical significance was calculated using a one-tailed or double-tailed t-test, depending on the case (GraphPad Prism 7 Software, San Diego, CA, USA). A value of *p* < 0.05 was considered statistically significant. The correlation between radiant efficiencies of fluorescently labelled nanoemulsions (measured by IVIS instrument) and naringenin content (determined by UHPLC) in organs from mice in experimental groups receiving either Nar/ICG/LN or V-Nar/ICG/LN was estimated using Pearson (r) and Spearman (ρ) correlation coefficients (GraphPad Prism 7 Software). For comparison, ρ and r were calculated with data from both experimental groups.

## 3. Results

### 3.1. Physico-Chemical Characterization of Naringenin/ICG-Loaded Nanoemulsions

Since naringenin lacks spectral properties, to follow the localization of i.v. administered nanoemulsions by fluorescence imaging, the NIR compound ICG was co-incorporated in the naringenin-loaded nanoemulsions formulations (Figure 1A). The lipid nanoemulsions were characterized by size and zeta potential. The values for naringenin-loaded nanoemulsions were recently reported elsewhere [18]. Herein, the results for the nanoemulsions incorporating both naringenin and ICG are presented. The table in Figure 1B summarizes the sizes, polydispersity indices (PDIs), and zeta potentials of non-targeted and VCAM-1 targeted nanoparticles immediately after preparation, and after storage for 3 months at 4 °C. We found that the size of the non-targeted Nar/ICG/LN nanoparticles was around 230 nm, and it remained practically unchanged after 3-month storage at 4 °C. By comparison, the size of targeted V-Nar/ICG/LN nanoparticles was around 220 nm, just after production, and increased slightly by the end of the indicated period to ~230 nm, but the PDI maintained at 0.2, indicative of a homogenous sample. As for the zeta potential, values were close to approximately −40 mV for both types of nanoemulsions at the initial time point, as well as at the end of the interval. Thus, there was no significant difference between the zeta potential of non-targeted as compared with VCAM-1 targeted nanoparticles. Figure 1C depicts images of ICG-loaded nanoemulsions displaying a milky appearance with an additional green hue due to the dye (Nar/ICG/LN), as compared with the nanoemulsions without ICG (Nar/LN). Figure 1D,F are representative illustrations of nanoparticles dimensions distributions for the non-targeted (D), respectively VCAM-1 targeted (F) nanoemulsions assessed at the initial and final time points. The zeta potential appeared as a sharp peak at both time points for non-targeted (Figure 1E), and targeted (Figure 1G) nanoparticles.

### 3.2. VCAM-1 Targeted Naringenin/ICG-Loaded Nanoemulsions (V-Nar/ICG/LN) Localize Significantly Higher in the Heart and Aorta of Mice with LPS-Induced Inflammation than the Non-Targeted Counterparts

**Figure 2:**
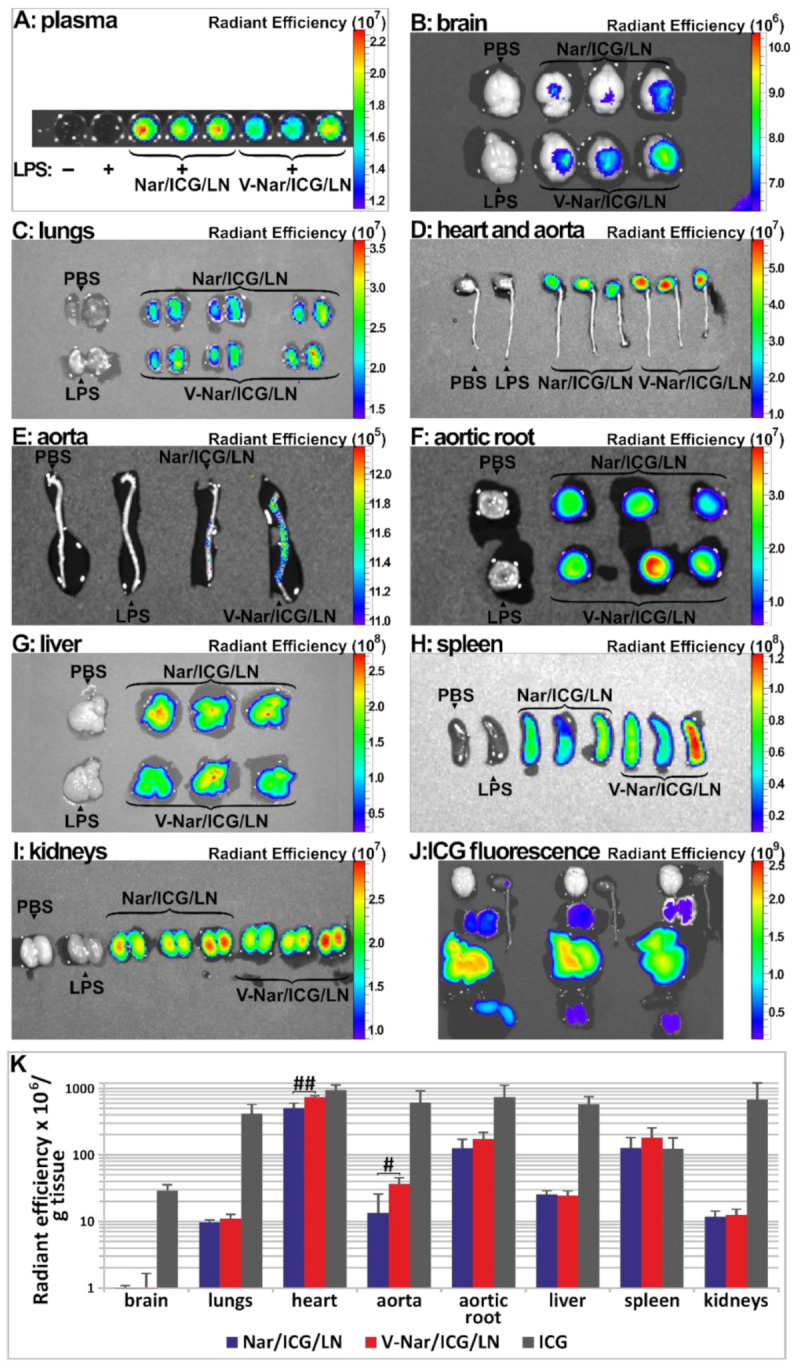
(**A**–**I**). Localization of ICG-labelled naringenin-loaded lipid nanoemulsions, either non-targeted (Nar/ICG/LN) or VCAM-1 targeted (V-Nar/ICG/LN), or of free ICG (ICG) in organs harvested from C57BL/6 mice. Measurements were recorded 1 h after retroorbital administration in a model of LPS-induced inflammation with a IVIS Spectrum imaging system Caliper 200, by detection of ICG fluorescence λex = 745 nm and λem = 820 nm. (**A**) plasma; (**B**) brain; (**C**) lungs; (**D**) heart and aorta; (**E**) aorta; (**F**) aortic root ; (**G**) liver; (**H**) spleen; (**I**) kidneys; (**J**) organs from animals in the free ICG group; (**K**) Comparative inter-organ quantification of fluorescence based on region-of-interest function of Living Image software; the values are shown as radiant efficiency per tissue weight (g); # *p* < 0.05, ## *p* < 0.01.

Taking into account that ICG did not affect the size, the stability, and the naringenin loading efficiency of the nanoparticles, it was used as a fluorescent probe to monitor the localization of i.v. administered nanoemulsions. To verify whether the VCAM-1 targeted, naringenin-loaded nanoparticles were specifically delivered towards the inflamed endothelium in vivo, an LPS-induced inflammation mouse model was used. Briefly, at 4 h after LPS administration, non-targeted (Nar/ICG/LN) or targeted (V-Nar/ICG/LN) nanoemulsions were administered retro-orbitally to C57BL/6 mice. After one hour, several organs were harvested from the mice (brain, lungs, heart and aorta, liver, spleen, and kidneys) and the nanoparticle localization was monitored, ex vivo, by detecting the NIR fluorescence of ICG in an IVIS Caliper System (Figure 2A–I).

To calculate the values of net radiant efficiency, the autofluorescence background represented by the average of values for PBS and LPS animals was subtracted from the raw signal and the comparative distribution over all the investigated organs is presented, based on the net radiant efficiencies, in Figure 2K. The results revealed that for the heart and the aorta, there was a statistically significant increased accumulation of VCAM-1 targeted nanoemulsions (V-Nar/ICG/LN) as compared to non-targeted nanoemulsions (Nar/ICG/LN) (*p* < 0.05 and *p* < 0.01, respectively). Further, a higher radiant efficiency of aortic root of animals injected with V-Nar/ICG/LN as compared with those receiving Nar/ICG/LN was detected, although it did not reach the level of statistical significance. The other organs did not show a differential uptake of the two types of naringenin-loaded nanoemulsions.

Of note, the spleen displayed an important accumulation of the fluorescence, comparable with that of the aortic root. However, there was no statistical significance between the targeted and non-targeted lipid nanoemulsions groups.

At the same time, the IVIS captures of the organs from the animals that received free ICG indicated a non-specific distribution across the investigated organs (Figure 2J). They are presented separately as their fluorescence is best represented on a different scale (see also ICG group in Figure 2K).

### 3.3. Naringenin Distribution in Organs Harvested from Mice Treated with V-Nar/ICG/LN or Nar/ICG/LN Subsequent to LPS-Induced Inflammation

**Figure 3:**
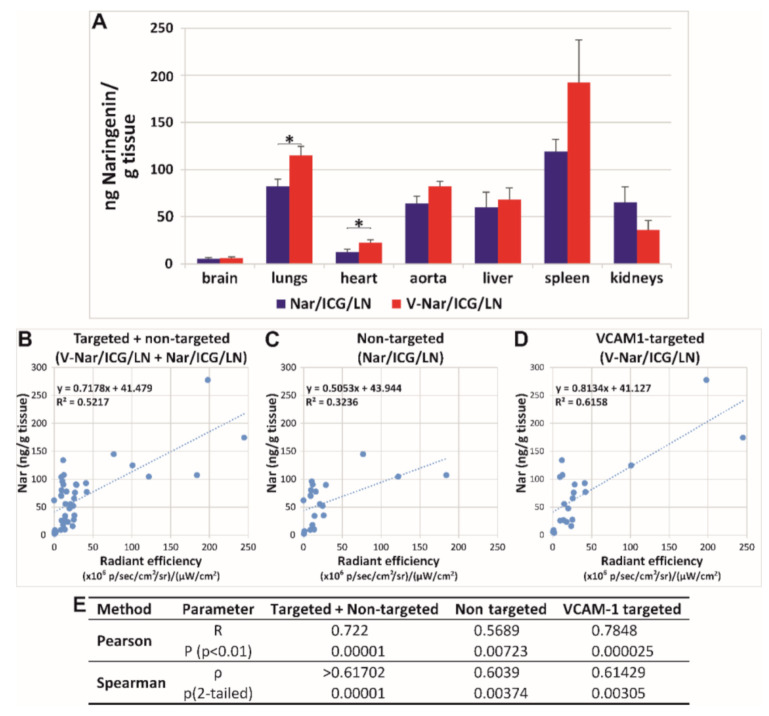
(**A**) UHPLC measurements of naringenin concentrations in the organs harvested from mice with LPS-induced inflammation at one hour after receiving intravenously ICG-labelled naringenin-loaded nanoemulsions, either non-targeted (Nar/ICG/LN) or VCAM-1 targeted (V-Nar/ICG/LN). Data represent the mean ± SD of values measured in organs isolated from 3 mice/experimental group; * *p* < 0.05. (**B**–**E**) Correlation between UHPLC measurements of naringenin content and fluorescent measurements of ICG radiant efficiency in organs from mice with LPS-induced inflammation. (**B**–**D**) Linear regression plots of naringenin content and radiant efficiencies in organs from mice in groups receiving (**B**) Nar/ICG/LN and V-Nar/ICG/LN**,** (**C**) Nar/ICG/LN, and (**D**) V-Nar/ICG/LN. (**E**) Results of correlation analysis by Pearson and Spearman methods.

To ascertain whether a correlation exists between the localization of ICG fluorescence and the naringenin content, the latter was quantified in homogenates of organs harvested from mice with LPS-induced inflammation and receiving V-Nar/ICG/LN or Nar/ICG/LN. The results of these measurements showed that the highest amount of Nar per gram of tissue was detected in the spleen, followed by the lungs, aorta, liver, kidney, heart, and brain (Figure 3B). Significant increases in the group receiving V-Nar/LN as compared with Nar/LN were detected in the heart (by ~50%, *p* < 0.05) and lungs (by ~40%, *p* < 0.05). Although increased concentrations of naringenin were also detected in the aorta in the case of VCAM-1 targeted LN as compared with non-targeted LN, the statistical significance was not achieved.

A correlation between the radiant efficiency measured by the IVIS instrument and naringenin content determined by UHPLC was sought. Linear regression generated r^2^ = 0.5217 when experimental points from organs of all mice in the groups Nar/ICG/LN and V-Nar/ICG/LN were plotted (Figure 3B); when the correlation was calculated separately for the two groups, r^2^ decreased to 0.3236 in the case of non-targeted LN (Figure 3C), yet it had a higher value of 0.6158 in the case of VCAM-1 targeted nanoparticles (Figure 3D). Although both Pearson and Spearman analyses indicated a moderate strength of correlation between the values obtained through fluorescence and UHPLC measurements (Figure 3E), graphical representation of the experimental data (Figure 3B–D) showed their scattering and thus cautioned on validating the correlation.

Overall, the amount of naringenin accumulated in all organs increased with 14% in the case of VCAM-1 targeted as compared with the non-targeted nanoparticles and the percentage distribution per organs is reported in Table 2. To assess whether there was a more efficient distribution of naringenin delivered by targeted V-Nar/ICG/LN as compared with Nar/ICG/LN, the percentage of naringenin in endothelium-rich organs versus the other organs was calculated, based on the formula (Nar_heart_ + Nar_lungs_)/(Nar_liver_ + Nar_kidneys_ + Nar_spleen_ + Nar_brain_). Values were 15.5 ± 2.1 for V-Nar/ICG/LN and 11.5 ± 1.5 for Nar/ICG/LN group, respectively, with a specific fold increase of 1.34 and a *p* value of 0.0312.

### 3.4. Naringenin-Loaded Nanoemulsions Exhibit an Anti-Inflammatory Effect in a Murine Model of Inflammation

To assess whether VCAM-1 targeted, naringenin-loaded nanoemulsions (V-Nar/LN) are functional and have anti-inflammatory effects in vivo, we employed the LPS-induced acute inflammation in mice as previously used in our laboratory [29]. Briefly, C57/BL6 mice received one retro-orbital injection, whereby LPS and the naringenin-loaded nanoemulsions were co-administered. After 24 h, several organs were harvested and the anti-inflammatory effects of V-Nar/LN were evaluated, measuring the gene expression of a representative panel of inflammatory markers: cytokines TNF-α and IL-1β, chemokines MCP-1 and RANTES, cell adhesion molecule VCAM-1, intercellular adhesion molecule (ICAM)-1, and platelet endothelial cell adhesion molecule (PECAM)-1.

**Figure 4:**
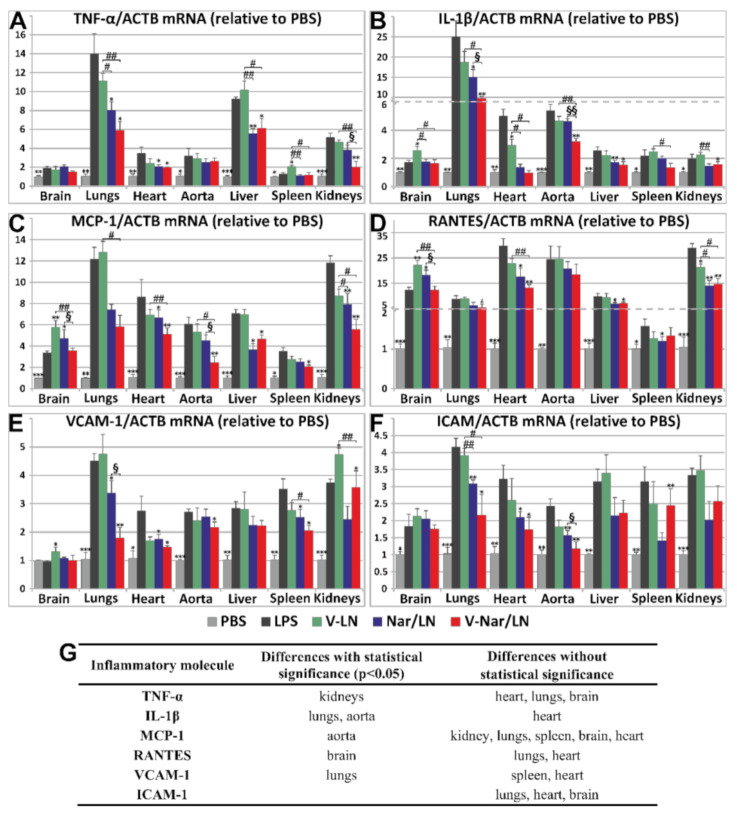
Gene expression of inflammatory markers (**A**) TNF-α, (**B**) IL-1β, (**C**) MCP-1, (**D**) RANTES, (**E**) VCAM-1, and (**F**) ICAM-1 in organs harvested from C57 BL/6 mice that were injected intravenously with non-targeted (Nar/LN) or VCAM-1 targeted (V-Nar/LN) naringenin-loaded nanoemulsions, administered retro-orbitally, in a murine model of LPS-induced inflammation. As controls, mice receiving PBS, LPS or VCAM-1 targeted empty nanoemulsions (V-LN) were used. Results were normalized to ACTB (β-actin) and are expressed as fold changes compared with the PBS-treated mice, considered as 1. The data are expressed as mean ± SD. Statistical significances: *, versus LPS; #, versus V-LN; §, versus Nar/LN; * *p* < 0.05, ** *p* < 0.01, *** *p* < 0.001; # *p* < 0.05, ## *p* < 0.01; § *p* < 0.05, §§ *p* < 0.01. There were 3 animals per group, except Nar/LN which consisted of 4 animals. (**G**) Summary of statistically significant reductions of the expression of LPS-induced inflammatory mediators, in different organs, by V-Nar/LN as compared to Nar/LN.

TNF-α gene expression was significantly increased by LPS treatment in all analyzed organs, with the fold changes relative to ACTB varying in the following order: lungs > liver> kidneys > heart ≈ aorta > brain > spleen (Figure 4A). The empty nanoemulsions (V-LN) did not affect supplementarily TNF-α gene expression as compared with LPS, except for the spleen, where the increase in TNF-α expression was significant (*p* < 0.05). Significant reductions of TNF-α expression by both Nar/LN and V-Nar/LN as compared with LPS were recorded in the lungs, heart, and liver. However, the only organ where a significant difference between the effects of non-targeted and targeted nanoemulsions occurred was the kidneys (*p* < 0.05).

IL-1β gene expression was significantly increased by LPS treatment in all analyzed organs, with the highest increase found in the lungs (~25-fold increase) as compared to controls (PBS-treated animals). After 24 h, the fold changes of IL-1β mRNA expression relative to ACTB showed the following order: lungs>> heart ≈ aorta > liver ≈ spleen ≈ kidneys > brain (Figure 4B). The empty nanoemulsions (V-LN) did not exert a significant effect as compared with LPS, apart from the brain and the heart where there was a supplementary increase of ~50% (*p* < 0.05) and a significant decrease of ~40% (*p* < 0.05), respectively, as compared with the LPS group. There were significant reductions of IL-1β gene expression, by both Nar/LN and V-Nar/LN in the liver and lungs as compared with LPS group and in the heart as compared with the V-LN group. Most importantly, V-Nar/LN exerted a significant effect (compared with Nar/LN) in reducing IL-1β gene expression in the lungs (*p* < 0.05) and aorta (*p* < 0.01).

MCP-1 gene expression was significantly increased by the treatment with LPS in all analyzed organs; after 24 h the fold changes relative to ACTB decreased in the order: lungs≈ kidneys > heart > liver > aorta> spleen ≈ brain (Figure 4C). The empty nanoemulsions (V-LN) did not have a significant effect as compared with LPS, with the exception of the brain, where there was a 1.7-fold increase (*p* < 0.05). In the kidneys, heart, and liver, both the non-targeted (Nar/LN) and targeted (V-Nar/LN) nanoemulsions significantly reduced MCP-1, as compared with the LPS group. Of note, VCAM-1 targeted naringenin-loaded nanoemulsions were significantly more effective in reducing MCP-1 gene expression in aorta (*p* < 0.05).

RANTES gene expression was significantly increased by the LPS treatment in all the analyzed organs; after 24 h the fold changes relative to ACTB varied in the following direction: heart ≈ aorta ≈ kidneys > brain ≈ liver ≈ lungs > spleen (Figure 4D). The empty nanoemulsions (V-LN) did not have a significant effect as compared with LPS, with the exception of the kidneys, where a ~25% decrease was detected (*p* < 0.05), and of the brain, where there was a 45% increase (*p* < 0.01). In heart, liver, and kidneys, both the non-targeted and the targeted nanoemulsions reduced RANTES gene expression as compared with the LPS group. Interestingly, V-Nar/LN reduced more pronouncedly RANTES gene expression as compared to non-targeted LN in the brain (*p* < 0.05).

VCAM-1 gene expression was significantly increased by LPS treatment in all the analyzed organs, after 24 h the fold changes relative to ACTB decreased in the series: lungs > kidneys ≈ spleen >liver ≈ heart ≈ aorta, except the brain (Figure 4E). The empty nanoemulsions (V-LN) additionally increased VCAM-1 as compared with LPS, with ~25% in the kidneys (*p* < 0.05) and with ~30% in the brain (*p* < 0.05). In the liver and brain, naringenin-loaded nanoemulsions did not significantly affect VCAM-1 gene expression. Both types of naringenin-loaded nanoemulsions reduced VCAM-1 as compared with the LPS group (*p* < 0.05) in the heart and spleen, and in the kidneys the reductions attained a higher significance (*p* < 0.01). In the lungs, VCAM-1 was reduced as compared with LPS group, by 25% in the case of Nar/LN, and by 40% in the case of V-Nar/LN, and the difference between the effect of the two formulations was significant (*p* < 0.05).

ICAM-1 gene expression was significantly raised by the LPS treatment of the mice in all the analyzed organs. After 24 h. the fold change relative to ACTB varied in the following order: lungs > kidneys ≈ heart ≈ spleen ≈ liver > aorta > brain (Figure 4F). In all analyzed organs, the empty emulsions had no significant effect as compared with the LPS group. A statistically significant reduction in ICAM-1 mRNA expression (compared to LPS-treated group) was obtained for both targeted and non-targeted naringenin-loaded LN in the lungs, heart, and aorta.

In the experimental model used herein, there were no significant changes of PECAM-1 gene expression upon treatment with LPS in the absence or the presence of Nar-loaded nanoemulsions (Supplementary Figure S6). This result is in good agreement with the published data, as PECAM-1 expression seems not to be affected by inflammation [30].

## 4. Discussion

Numerous major current pathologies, including cardiovascular disease [31], cancer [32], diabetes [33], and neurodegeneration [34] are associated with a chronic inflammatory process. As an alternative to synthetic anti-inflammatory compounds, natural compounds with less toxicity and well-tolerated profiles are envisaged for treatment. Polyphenols, the largest class of natural compounds, are intensely investigated due to their therapeutic potential [35]. The poor hydrosolubility and bioavailability of many members of this class stimulated the development of their formulations in nanocarriers [36]. The development of theranostic nanoparticles, which combine a nanocarrier for a therapeutic agent with an imaging probe for the non-invasive monitoring of the in vivo biodistribution, is one of the essential goals of bionanotechnologies [37]. Inducible adhesion molecules expressed specifically on the endothelial surface upon activation by inflammatory stimuli are attractive targets for the delivery of imaging and therapeutic agents encapsulated in nanoparticles [38].

We have previously reported that VCAM-1 targeted naringenin-loaded lipid nanoemulsions (LN) have the capacity to reduce endothelial inflammation in vitro [18]. In this study, to investigate the biodistribution of this flavonoid in vivo, an original and novel nanoformulation was designed based on loading LN with naringenin and the NIR probe, ICG. As compared with nanoemulsions loaded only with naringenin (Nar/LN), previously prepared by a similar procedure and with the same lipid composition [18], we found no major changes in the dimensions of the LN (~200 nm) and the efficiency of flavonoid loading (~80%) upon ICG co-incorporation. Moreover, the incorporation of ICG in the newly designed LN (Nar/ICG /LN) did not affect the zeta potential of targeted and non-targeted LN that displayed similar values of ~−40 mV. Previously, we showed that coupling of the VCAM-1 recognition peptide to naringenin-loaded nanoemulsions shifted the zeta potential towards negative values, proportionally with the amount of coupled peptide [18]. Herein, the comparable zeta potential of both non-targeted and targeted LN could be explained by the mode ICG inserts into the micelle, with the uncharged moiety penetrating the lipid monolayer, while the negatively charged, hydrophilic part that cannot be accommodated into the hydrophobic core of the LN remains oriented outwards. The SO3^-^ charge buffers the positive charge of the VCAM-1 recognition peptide and consequently, this no longer attracts supplementary negative charges to shift the zeta potential. This explanation is also supported by the reported data showing by NMR that ICG encapsulated in lipid nanoparticles was distributed closer to the surface by contrast with DiI, a more hydrophobic fluorescent probe that inserts more profoundly [39]. In addition, we found that after three-month storage at 4 °C, in the dark, both the non-targeted and targeted nanoemulsions maintained the dimensions and zeta potential practically unchanged. This behavior indicated a good stability and recommends this formulation for preserving ICG properties, as this molecule is susceptible to degradation in aqueous solutions [40]. Further, the experiments performed in the presence of plasma indicated that both Nar/LN and Nar/ICG/LN are stable, displaying unchanged size, PDI, and zeta potential (Supplementary Table S1).

The studies performed on experimental inflammation in mice revealed a significantly increased accumulation of VCAM-1 targeted, naringenin-loaded, and ICG-labeled LN (V-Nar/ICG/LN) in the heart and aorta as compared to non-targeted counterparts. Could our data be an artefact due to a preferential localization of ICG to inflamed sites of the heart and aorta? Data from the literature support the fact that ICG has a strong affinity for plasma proteins and lipids [41,42], as well as a tropism for the lipid-rich inflamed regions of the cardiovascular segment [21]. Taking into account that both targeted and non-targeted nanoparticles were similar regarding the dimensions and zeta potential, as well as the ICG content, we assumed that the differential interaction with the inflamed endothelium of the two types of nanoparticles was mainly due to the presence on the surface of the targeted nanoparticles of the VCAM-1 recognition peptide. Additionally, the experimental group that received free ICG presented a relatively non-specific distribution, as demonstrated by the amounts accumulated in the aortas, aortic roots, livers, kidneys, and lungs.

Apparently, these findings may contradict the idea that free ICG is rapidly eliminated from circulation through the hepato-biliary pathways. Yet, the study of ICG administered intravenously in pig showed that ICG biodistribution was more accurately described by a temporary redistribution model than by the single pass model, explaining the detection of significant amounts in the extravascular and extrahepatic space in organs such as kidneys, lungs, spleen [43]. In addition, it was shown that a 45-min treatment with LPS in rats decreased the rate of hepatic uptake of ICG [44], results that also agree with our data showing similar amounts of ICG in liver, kidneys, and lungs. 

To confirm our data, we evaluated the naringenin content in the harvested organs. As indicated in the Results section, a modest correlation between fluorescence and UHPLC data was established, yet a sound validation cannot be claimed. There are a series of causes that may have contributed to this disagreement.

First, there are certain limitations associated with fluorescence measurements. In fact, imaging techniques for analyzing the biodistribution of nanoparticles may be used mostly as a semi-quantitative tool, as they may be affected by the saturation of the signal, fluorescence quenching at high concentrations of the fluorophore as it accumulates at a particular site, and differential photobleaching of the probe in organs of dissimilar width [45].

In the current report, a significant issue may be represented by the alteration of the photochemical stability, and subsequently, of the quantum yield of ICG by the microenvironment ([46,47], Supplementary Figure S5). Thus, it must be reinforced that the comparison of raw radiant efficiencies data between the group of mice that received free ICG and the groups that received ICG-labeled nanoemulsions does not reflect a real ratio between the accumulations of ICG in the two conditions. On one hand, in the circulation, plasma proteins bind free ICG, increasing its fluorescence. On the other hand, ICG incorporated in nanoparticles establishes physical contacts with constituent phospholipids. In addition, another assumption underlying our interpretation was that both the non-targeted and targeted nanoparticles emit equivalent signals at equal accumulation, but this could be inaccurate, as the two LN types may adsorb different coronas.

Further, the release of the fluorescent probe from the nanoparticles should proceed with slow kinetics, reducing the possibility of false results. It is known that ICG has a peculiar avidity for plasma proteins, and these were capable to extract ICG from lipid nanoparticles where it was partially exposed on the surface [39]. For this, the measured radiant efficiency at a certain moment in vivo may be a sum of contributions from ICG bound by plasma proteins and/or lipids and ICG incorporated in LN, a fact that could explain certain discrepancies between the fluorescence data obtained by IVIS evaluation and the analytical measurements of naringenin by UHPLC. Although in our experiments we assessed in vitro the stability of ICG-loaded LN in the presence of plasma (Supplementary data, Table S1 and Figure S2), this may not thoroughly extrapolate to the in vivo conditions, in the presence of the actual hemodynamic stress.

Secondly, regarding the UHPLC measurements, the method utilized detected only free naringenin, as present in nanoemulsions. However, it is known that naringenin in vivo undergoes rapid metabolic changes into glucuronides and sulfates [48], and, for the quantification of these derivatives, two additional sets of measurements, involving pre-treatment with β-glucuronidase and respectively sulfatase, would have been needed [49].

According to the fluorescence measurements, the accumulation of targeted V-Nar/ICG/LN decreased in the order: spleen ≈ aortic root > aorta > heart ≈ liver > kidney > lungs > brain. In the same time, HPLC data for naringenin quantification indicated a variation in the series: spleen > lungs > aorta ≈ liver > kidney > heart > brain. Thus, by both methods, fluorescence and HPLC, the spleen is a main site for naringenin-loaded LN accumulation. This agrees with the data from the literature, which show that nanoparticles >200 nm accumulate particularly in the spleen, both in physiological conditions [50,51], and especially in the case of systemic inflammation [52], such as that induced by LPS. Furthermore, an important issue to be emphasized is that, when orally administered in rats, naringenin localizes most in gastrointestinal tract, liver, kidney, lungs, and trachea, but not in the spleen [53]. The detection of large amounts of naringenin in the spleen supports the local accumulation of the nanoemulsions, as detected by fluorescence, at this site.

Analysis of the fluorescence and HPLC data led to the conclusion that the pharmacokinetics of targeted nanoparticles that co-encapsulate naringenin and ICG is the result of a mixed behavior. On one hand, there is a passive accumulation, driven by the LPS-induced increased vascular permeability in spleen, kidneys, and liver and, on the other hand, there is the targeted delivery towards inflamed sites based on VCAM-1 recognition. While the renal and spleen accrual of naringenin may be attributed primarily to renal and splenic filtration, the accumulation in the heart and lungs is due predominantly to targeted delivery. This is not surprising since these tissues are endowed with a particularly high number of endothelial cells that are activated upon exposure to LPS.

At this point, a question may be raised regarding the benefits brought by the targeted nanoparticles. Our results demonstrated a modest gain of the overall accumulation, across all organs, of naringenin delivered by the VCAM-1 directed nanoemulsions, and increases were detected in the spleen, heart, and lungs. Further, there was a reduction in the renal accumulation of naringenin, that may reflect a retarded excretion. Overall, the results demonstrated a small but significant (*p* < 0.05) targeting effect in the case of VCAM-1 targeted nanoparticles, and it may support the development of this nanoformulation. Analyzing the data from the real-time PCR experiments, it became apparent that naringenin loaded-nanoparticles, both non-targeted (Nar/LN), and targeted (V-Nar/LN) reduce the inflammatory markers, as detailed in the Results section. Statistical significances for a more potent effect of V-Nar/LN as compared with the non-targeted Nar/LN were recorded in the brain (for RANTES), lungs (for IL-1β and VCAM-1), aorta (IL-1β and MCP-1), and kidneys (for TNF-α). It can be noticed that, in the lungs, where both IL-1β and VCAM-1 were reduced more by V-Nar/LN in comparison with Nar/LN, there was also a significant amount of naringenin accumulated in a manner dependent on the targeting of VCAM-1. These data strengthen the targeting potential of the peptide that recognizes VCAM-1 in the lungs. This was previously validated through the efficient delivery of a CCR2 antagonist encapsulated in liposomes targeted to VCAM-1, with anti-metastatic effects on pulmonary tumors in a murine model, as well as on human samples [54]. On the other side, there are numerous studies that proved molecular mechanisms of naringenin in reducing pulmonary inflammation, both acute [55] and chronic [15,56], in animal experimental models [57,58], as well as in human subjects [59]. These data have already started to be translated in therapeutic strategies with clinical applications, by the preparation of phytosomes based on dipalmitoyl phosphatidylcholine loaded with naringenin for pulmonary delivery by inhalation [60]. Moreover, our data are in line with and extend the reports showing that naringenin decreases IL-1β expression in irradiation-induced injury [61] and fibrosis post-infection with Mycoplasma pneumoniae [59]. In our studies, we also assessed the TNF-α gene expression. Our data indicated a significant reduction in TNF-α in the kidneys of animals with LPS-induced inflammation treated with naringenin-loaded nanoemulsions, with a higher reduction in its level in the case of targeted V-Nar/LN. These data align with the previously reported results showing that TNF-α was involved in inflammatory conditions such as acute renal injury [62,63], and naringenin prevented the renal toxicity induced by doxorubicin in Wistar rats [64].

## 5. Conclusions

To conclude, the novel data of this study are: (1) the development of ICG-labelled, naringenin-loaded nanoemulsions to be employed in monitoring the biodistribution of the polyphenol following i.v. administration; (2) introduction of the VCAM-1 targeted, ICG-labelled naringenin-loaded nanoparticles as a potential delivery platform to the heart and lungs of mice with LPS-induced inflammation; (3) demonstration that naringenin-loaded nanoemulsions are functional and that their therapeutic effects involve, to some extent, a reduction in inflammatory markers in experimental inflammation. In this study, the anti-inflammatory effects of naringenin-loaded LN were assessed based on the reduction in the gene expression of inflammatory molecules, as this was considered to best fit the time frame of our experimental set-up. Yet, other mechanisms, such as post-translational events, may participate in the reduction in the inflammatory response. For these reasons, a complementary evaluation of the therapeutic effect of naringenin could be pursued.

VCAM-1 targeted naringenin-loaded lipid nanoemulsions may serve as a candidate nanoplatform for theranostic applications in systemic inflammation. A statistically significant higher localization of naringenin delivered by VCAM-1 targeted nanoemulsions takes place in the heart and lungs of mice subjected to LPS-induced inflammation compared to non-targeted counterparts. The mechanism underlying the therapeutic potential of naringenin-loaded lipid nanoemulsions consists, at least in part, in the decreased expression of inflammatory molecules in experimental inflammation.

Further studies may contribute to the improvement of this nanoformulation as a theranostic tool through a more detailed, time-course investigation of its fate in vivo by correlating the fluorescence data with the HPLC assessment of both ICG and encapsulated drug content in organs. These investigations should also encompass other anti-inflammatory agents to extend the potential application of ICG labeled nanoemulsions in targeted drug delivery.

## Figures and Tables

**Table 1:** Sequences of the primers for the murine genes whose expression was assessed in real time qPCR experiments.

Gene	Ref Seq	Forward	Reverse	Amplicon (bp)
**ACTB**	NM_007393.5	GACGAGGCCCAGAGCAAGAGAGG	CATGGCTGGGGTGTTGAAGGTCTC	231
**RANTES**	NM_013653.3	GACACCACTCCCTGCTGCTTTG	CACACACTTGGCGGTTCCTTCG	136
**MCP-1**	NM_011333.3	AAGAAGCTGTAGTTTTTGTCACC	CAGATTTACGGGTCAACTTCACA	275
**TNF-α**	NM_013693.3	GAGGTCAATCTGCCCAAGTA	GTAGAGAATGGATGAACACCC	100
**IL1beta**	NM_008361.4	CAACCAACAAGTGATATTCTCCA	TCTTTCATTACACAGGACAGGT	117
**VCAM-1**	NM_011693.3	ATTATCCAAGTCTCTCCAAAAG	TGTCTTTGCTTTCTTCTTCAGGA	141
**ICAM-1**	NM_010493.3	GGTTCTTCTGAGCGGCGTCG	CCAGCCGAGGACCATACAGC	179
**PECAM-1**	NM_001305158	ATTACGGTTATGATGATGTTTCTGG	CCGTCTCTGTGGCTCTCGTTC	150

**Table 2:** Percentages of naringenin detected by UHPLC in the organs harvested from mice with LPS-induced inflammation at one hour after receiving intravenously ICG-labelled naringenin-loaded nanoemulsions, either non-targeted (Nar/ICG/LN) or VCAM-1 targeted (V-Nar/ICG/LN). Percentages were calculated relative to the total amount of naringenin in all organs for each experimental group. Data represent the mean ± SD of values for 3 mice/experimental group.

Nanoparticle	Organ						
	Brain	Lungs	Heart	Aorta	Liver	Spleen	Kidneys
V-Nar/ICG/LN	2.7 ± 0.9	22.6 ± 2.1	2.2 ± 0.7	0.7 ± 0.1	49.6 ± 8.2	15.7 ± 6.2	6.6 ± 2.7
Nar/ICG/LN	2.8 ± 1.4	19.5 ± 4.6	1.5 ± 0.5	0.8 ± 0.4	49.5 ± 12.1	11.1 ± 2.4	14.8 ± 7.4

## Supplementary Materials

The following are available online at https://www.mdpi.com/1999-4923/12/11/1066/s1. Figure S1. Optical microscopy images of Nar/LN (A), V-Nar/LN (B), Nar/ICG/LN (C) and V-Nar/ICG/LN; Figure S2. The variations of the intensity distributions and zeta potentials of lipid nanoemulsions loaded with ICG (ICG/LN), naringenin (Nar/LN) or both (Nar/ICG/LN) after incubation for 1 h in the presence of plasma at 37 °C). Upper panels correspond to measurements of hydrodynamic diameters, and bottom panels-to measurements of zeta potential. The black curves represent the initial moment, and the green ones, the final moment. In the samples marked LN+Nar, LN+ICG, LN+Nar+ICG, naringenin and ICG were added, at the corresponding concentrations, from stock solutions to empty nanoemulsions (LN); Figure S3. Radiant efficiency of ICG as a fluorescent probe for the naringenin-loaded nanoemulsions. Data are graphical representations of the radiant efficiencies of nanoemulsions samples with increasing concentrations of ICG at the initial time (to) and after storage for 24 h at room temperature; Figure S4. Evaluation of radiant efficiency of ICG (A, C, E) as compared with Rhodamine-PE (B, D, F), maintained in the dark for 24 h in the absence or presence of FBS in various microenvironments: fluorophores as free substances in PBS (A, B), added to pre-formed nanoemulsions (C, D), or stably incorporated into nanoemulsions (E, F); Figure S5. Radiant efficiency of ICG (A, C) compared with Rhodamine (B, D) as a fluorescent probe for the naringenin-loaded nanoemulsions. The radiant efficiency of naringenin-loaded nanoemulsions labeled with ICG or Rhodamine-PE incubated in 50% FBS in PBS was measured with an IVIS Spectrum Caliper 200 system. The upper panels (A, B) illustrate the radiant efficiencies of ICG, respectively Rhodamine for the indicated concentrations at the initial time point. The lower panels (C, D) depict graphical representations of the radiant efficiencies of naringenin-loaded nanoemulsions with increasing concentrations of the fluorescent probe at the initial time (T_0_) and after storage for 24 h at room temperature; Figure S6. Gene expression of PECAM-1 in organs harvested from C57 BL/6 mice that were injected intravenously with non-targeted (Nar/LN) or VCAM-1 targeted (V-Nar/LN) naringenin-loaded nanoemulsions, administered retro-orbitally, in a murine model of LPS-induced inflammation. As controls, mice receiving PBS, LPS or VCAM-1 targeted empty nanoemulsions (V-LN) were used. Results were normalized to ACTB (β-actin) and are expressed as fold changes compared with the PBS-treated mice, considered as 1. The data are expressed as mean ± SD. There were 3 animals per group, except Nar/LN which consisted of 4 animals; Table S1. Summary of hydrodynamic diameters, polydispersion indices (PDI) and zeta potentials for naringenin-loaded lipid nanoemulsions in the presence of murine plasma, at the initial (to) and final (1 h) time points. In the samples marked LN+Nar, LN+ICG, LN+Nar+ICG, naringenin and ICG were added, at the corresponding concentrations, from stock solutions to empty nanoemulsions (LN); Table S2. Radiant efficiencies (per g tissue), based on ICG fluorescence, of the organs from mice with LPS-induced inflammation, which received non-targeted (Nar/ICG/LN) or VCAM-1 targeted (V-Nar/ICG/LN) nanoemulsions, at one-hour post-injection. Control (of autofluorescence) is represented by PBS- and LPS-injected animals.
