# Supplementary material for: Evaluation of VCAM-1 Targeted Naringenin/Indocyanine Green-Loaded Lipid Nanoemulsions as Theranostic Nanoplatforms in Inflammation
Source: Pharmaceutics. 2020 Nov 9;12(11):1066. doi: 10.3390/pharmaceutics12111066 (PMC7695317; doi:10.3390/pharmaceutics12111066)

Size Distribution Report by Intensity

v2.2

Sample Details

Sample Name: V-Nar/ICG/LN (T0)

SOP Name: LN_EF_apa.sop

General Notes:

**File Name:**	no42.dts	**Dispersant Name:**	Water
**Record Number:**	240	**Dispersant RI:**	1.330
**Material RI:**	1.45	**Viscosity (cP):**	0.8872
**Material Absorbtion:**	0.001	**Measurement Date and Time:**	Thursday, 18 July, 2019 08:...

System

**Temperature (°C):**	25.0	**Duration Used (s):**	60
**Count Rate (kcps):**	369.6	**Measurement Position (mm):**	4.65
**Cell Description:**	Disposable sizing cuvette	**Attenuator:**	8

Results

			Size (d.n...	% Intensity:	St Dev (d.n...
**Z-Average (d.nm):**	217.2	**Peak 1:**	249.1	100.0	104.2
**PdI:**	0.163	**Peak 2:**	0.000	0.0	0.000
**Intercept:**	0.924	**Peak 3:**	0.000	0.0	0.000

Result quality **Good**

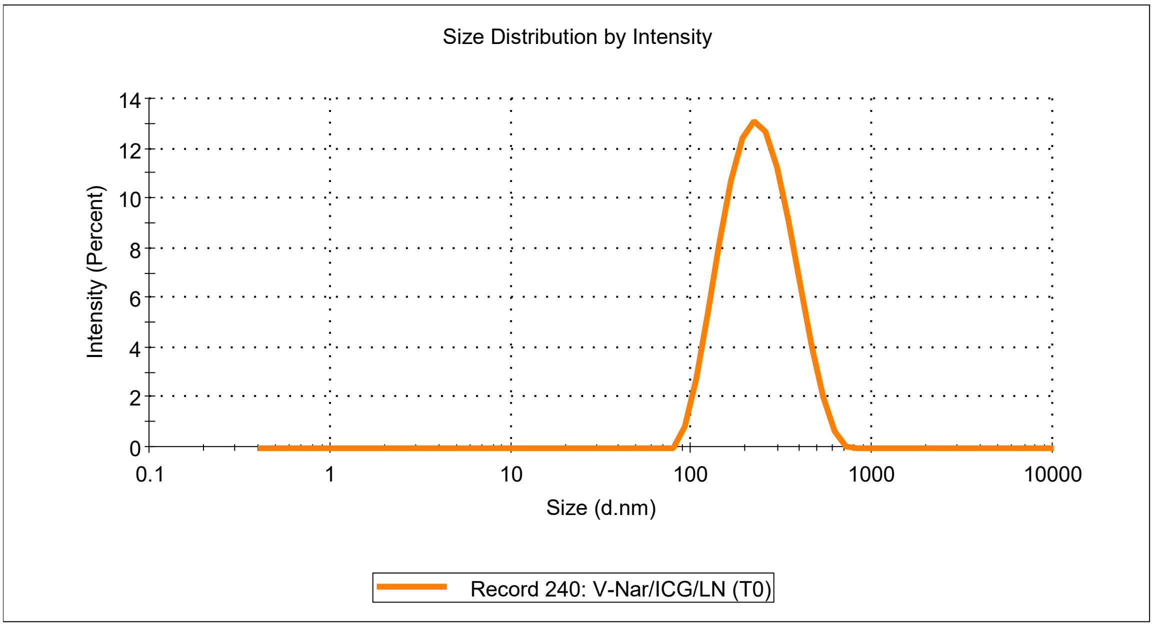

Supplement: Supplementary file 1 [file pharmaceutics-12-01066-s001.zip › pharmaceutics-976344-sup/Fig 1 VNar ICG LN T0.pdf]
