# Supplementary material for: Evaluation of VCAM-1 Targeted Naringenin/Indocyanine Green-Loaded Lipid Nanoemulsions as Theranostic Nanoplatforms in Inflammation
Source: Pharmaceutics. 2020 Nov 9;12(11):1066. doi: 10.3390/pharmaceutics12111066 (PMC7695317; doi:10.3390/pharmaceutics12111066)
Supplement: Supplementary file 1 [file pharmaceutics-12-01066-s001.zip › pharmaceutics-976344-sup/pharmaceutics-976344-Supplementary_revMC.docx]

Article

Supplementary Materials: Evaluation of VCAM-1 Targeted Naringenin/ Indocyanine Green-Loaded Lipid Nanoemulsions As Theranostic Nanoplatforms In Inflammation

Elena Valeria Fuior, Cristina Ana Mocanu, Mariana Deleanu, Geanina Voicu, Maria Anghelache, Daniela Rebleanu, Maya Simionescu, Manuela Calin

1. Optical microscopy of naringenin/ ICG-loaded nanoemulsions

Optical microscopy was performed using an inverted Olympus IX81 microscope equipped with 60x objective with 1.6x magnifying lens. One drop of nanoemulsion was placed between a microscope slide and a cover slip. Photographs were acquired in the same exposure conditions using CellSens software and processed with ImageJ freeware. No large aggregates were detected and particles were relatively uniform in size (**Figure S1)**.


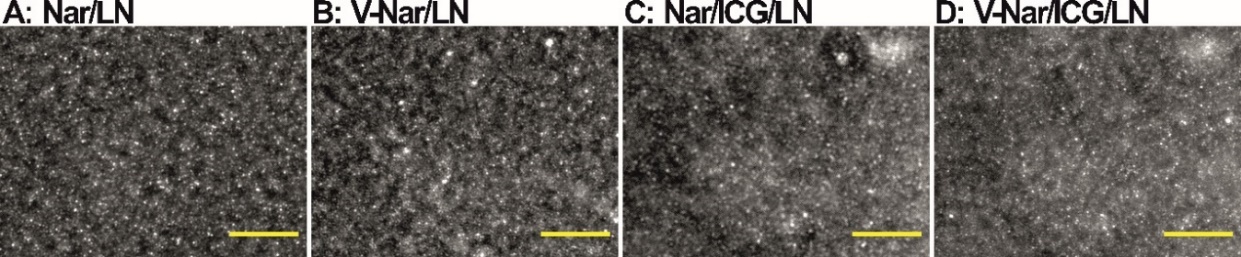


**Figure S1.** Optical microscopy images of Nar/LN (A), V-Nar/LN (B), Nar/ICG/LN (C) and V-Nar/ICG/LN. Scale bar: 20 μm.

At the end of the Supplementary material section two Size Distribution Reports can be found, as generated by Malvern Zetasizer instrument for size of Nar/ICG/LN and V-Nar/ICG/LN at the time of preparation. No particles can be detected with size > 1 μm, as the instrument upper limit detection is 10 μm.

2. Evaluation of naringenin-loaded lipid nanoemulsions stability

The stability of intravenously administered nanoemulsions is a key requirement to benefit from the therapeutic potential of targeted nanoparticles. To assess the stability of nanoemulsions loaded with naringenin and ICG in the presence of plasma *in vitro*, nanoemulsions were incubated with 50% murine plasma in PBS, on a rotative shaker for one hour at 37°C. The concentrations of the components of the mix were: 1mM total lipid, 300 µM naringenin (incorporated in the nanoemulsions or added from a stock solution), 15 µM ICG (incorporated in the nanoemulsions or added from a stock solution). These concentrations were established based on the ratio between the volume of the nanoemulsions and the total blood volume of a mouse, as occurred upon i.v. administration in the murine inflammation model. After 1 h, the samples were centrifuged on Amicon columns at 16000 *g* for 5 min, and naringenin was quantitated both in the filtrate and the concentrate by UHPLC. The results indicated that for Nar/LN, 5.6% of the initial amount of naringenin was retrieved in the filtrate, but only 1.43% in the case of Nar/ICG /LN.

Also, the nanoemulsions were characterized by measuring the dimensions and zeta potential using the ZetaSizer NanoZS instrument (Malvern Instruments, UK) at the initial and final time points of incubation (**Table S1**). As controls, empty nanoemulsions (LN) were used, to which naringenin and/or ICG were added from stock solutions, in amounts equivalent to those incorporated in nanoemulsions.

**Table S1.** Summary of hydrodynamic diameters, polydispersion indices (PDI) and zeta potentials for naringenin-loaded lipid nanoemulsions in the presence of murine plasma, at the initial (t_o_) and final (1 h) time points. In the samples marked LN+Nar, LN+ICG, LN+Nar+ICG, naringenin and ICG were added, at the corresponding concentrations, from stock solutions to empty nanoemulsions (LN).

| **Sample** | **Size (nm)** | | **PDI** | | **Zeta potential (mV)** | |
| --- | --- | --- | --- | --- | --- | --- |
|  | **initial** | **final** | **initial** | **final** | **initial** | **final** |
| LN | 220.9 ± 0.3 | 220.2 ± 2.2 | 0.216 ± 0.004 | 0.223 ± 0.004 | −26.6 ± 1.6 | −35.5 ± 3.2 |
| Nar/LN | 235.6 ± 2.4 | 234.8 ± 2.9 | 0.217 ± 0.014 | 0.216 ± 0.024 | −42.9 ± 0.5 | −44.1 ± 1.0 |
| ICG/LN | 242.8 ± 3.0 | 234.9 ± 3.2 | 0.234 ± 0.012 | 0.208 ± 0.027 | −42.6 ± 0.7 | −40.0 ± 0.6 |
| Nar/ICG/LN | 241.1 ± 4.7 | 250.2 ± 2.5 | 0.226 ± 0.026 | 0.221 ± 0.031 | −36.7 ± 0.9 | −35.7 ± 0.7 |
| LN + Nar | 224.6 ± 1.2 | 222.4 ± 1.6 | 0.221 ± 0.010 | 0.215 ± 0.039 | −41.5 ± 2.0 | −42.3 ± 1.1 |
| LN + ICG | 211.2 ± 2.8 | 215.2 ± 0.4 | 0.251 ± 0.012 | 0.267 ± 0.012 | −42.7 ± 1.0 | −40.7 ± 0.3 |
| LN + Nar + ICG | 230.0 ± 0.3 | 222.5 ± 1.6 | 0.225 ± 0.015 | 0.283 ± 0.018 | −43.0 ± 1.2 | −41.2 ± 0.8 |

Data analysis indicated that there were no significant differences regarding the dimensions and PDIs. Regarding the zeta potential, the only significant alteration was noticed in the case of empty nanoemulsions (LN) for which the incubation for one hour in the presence of serum brought a shift from -26.6 to -35.5 mV. This fact could be due to the adsorption of plasma proteins on the surface of the nanoparticles. By examining the intensity distributions (**Figure S2**, upper panels) it can be noticed that addition of naringenin, alone or in combination with ICG, induced a change in the particle size of the empty nanoemulsions (LN), upon incubation with plasma, by an asymmetric shift towards the smaller dimensions. When ICG alone was added to the nanoemulsions, no change occurred. By contrast, the nanoemulsions which contain encapsulated Nar (Nar/LN), ICG (ICG/LN) or both compounds (Nar/ICG LN) were stable under the experimental set-up. Of note, no effects on zeta potential were noticed (**Figure S2**, bottom panels).


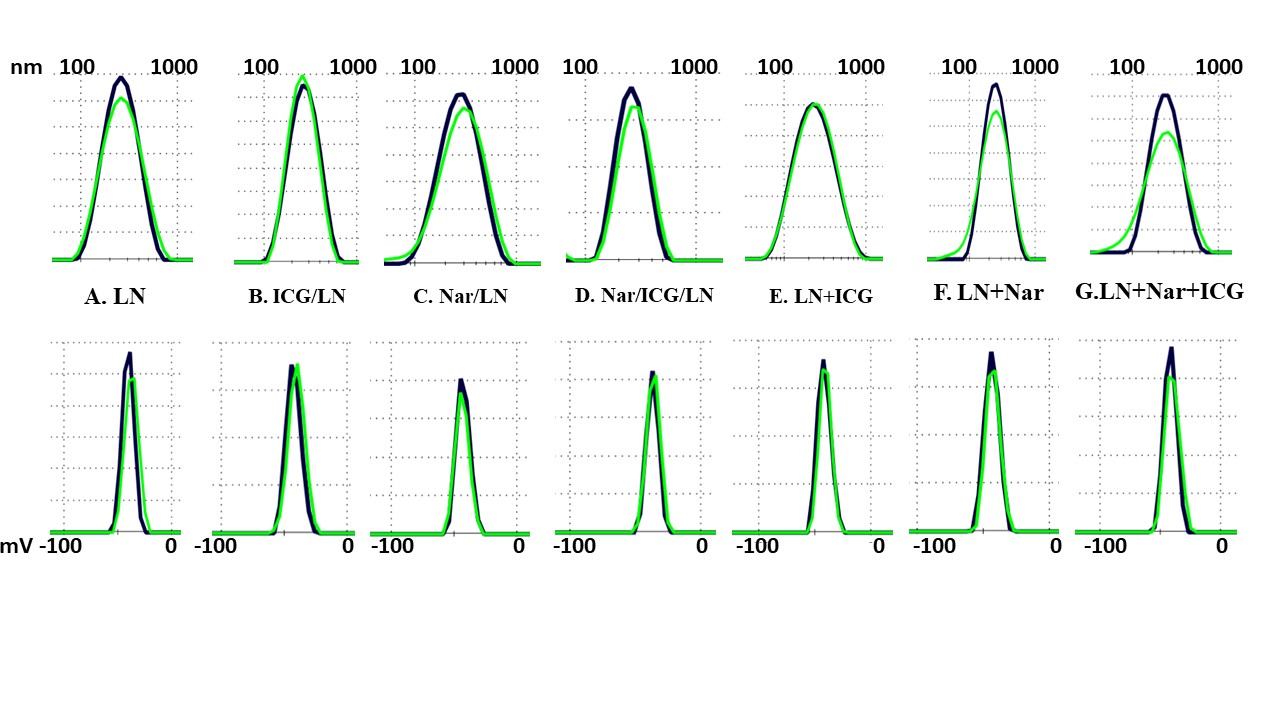


**Figure S2.** The variations of the intensity distributions and zeta potentials of lipid nanoemulsions loaded with ICG (ICG/LN), naringenin (Nar/LN) or both (Nar/ICG/LN) after incubation for 1 h in the presence of plasma at 37 ^o^C). Upper panels correspond to measurements of hydrodynamic diameters, and bottom panels to measurements of zeta potential. The black curves represent the initial moment, and the green ones-the final moment. In the samples marked LN+Nar, LN+ICG, LN+Nar+ICG, naringenin and ICG were added, at the corresponding concentrations, from stock solutions to empty nanoemulsions (LN).

The results indicated that incorporation of naringenin into nanoemulsions in the absence or presence of ICG (samples Nar/LN, respectively Nar/ICG/LN) is stable in the presence of plasma, whereas free naringenin added to empty nanoemulsions, alone (sample LN+Nar) or together with ICG (sample LN+Nar+ICG) tended to destabilize the nanoemulsions, due to its hydrophobicity and propensity to precipitate and/or incorporate in nanoparticles. Meanwhile, free ICG did not influence LN stability (sample LN+ICG), a fact that can be rationalized based on the electrostatic charge of ICG, that ensured a better hydrosolubility as compared with naringenin. When both substances with reduced water solubility were present, there was a supplementary reduction of the solubility of naringenin, as the least soluble of the two compounds, and thus the destabilizing effect of naringenin was amplified.

*3.* Evaluation of ICG efficiency as a fluorescent probe for naringenin-loaded nanoemulsions

We assessed the radiant efficiency of naringenin-loaded nanoemulsions labeled with ICG, at concentrations up to 210 µM, using IVIS Spectrum Caliper 200 system. We found that, both at the initial time point (t_o_) and after 24 h, the shape of the dose-response curve is biphasic, first ascending, then descending. We attributed this aspect to fluorescence quenching effects. The decay in the quantum yield of ICG over time would explain the shift to the right of the curve seen at 24 h (**Figure S3**).

**Figure S3.** Radiant efficiency of ICG as a fluorescent probe for the naringenin-loaded nanoemulsions. Data are graphical representations of the radiant efficiencies of nanoemulsions samples with increasing concentrations of ICG at the initial time (t_o_) and after storage for 24 h at room temperature.

To assess the changes of ICG quantum yield in various microenvironments, we measured its radiant efficiency in the free form (**Figure S4**, **A**), when added to pre-formed LN (**Figure S4**, **C**), or stably incorporated into nanoemulsions (**Figure S4**, **E**). Measurements were performed with the aid of the IVIS Spectrum Caliper 200 system at the initial moment or after 24-h storage at room temperature, in the absence of presence of fetal bovine serum (FBS). For comparison, samples containing Rhodamine -PE instead of ICG were also investigated (**Figure S4**, **B**, **D**, **F**).

The data indicated that in PBS, both ICG (**Figure S4, A**) and Rhodamine (**Figure S4, B**) barely displayed any fluorescence. The behavior upon FBS addition was different: for ICG, the fluorescence increased with one order of magnitude, with values of radiant efficiency of 10^9^, which maintained during the analyzed period (curve ICG+FBS), as for Rhodamine-PE the increase was virtually negligible.

When the substances were added to empty nanoemulsions (LN), an increase in the fluorescent signal occurred due to the hydrophobic environment. The behavior of ICG added to LN is complex (**Figure S4**, **C**). Thus, when adding ICG to LN, a peak was attained for 10 µM ICG, close to the value recorded in FBS, followed by a linear reduction of the fluorescence at concentrations higher than 20 µM. The curve profile was similar after 24 h, but with values higher as compared with the initial moment. In the presence of FBS, initially there was a 25% increase of fluorescence as compared with the case without FBS. After 24 h, the shape of the curve is changed to a hyperbola, this alteration reflecting the two different processes ICG participates in: on one hand, insertion in the lipid monolayer and, on the other hand, the association with plasma proteins. This partitioning decreases the effective concentration and the physical separation of the molecules, contributing to the reduction of quenching. For Rhodamine-PE added to LN, the profiles of the curves in presence or absence of FBS were similar (**Figure S4**, **D**). Initially there was a slight decrease, however 24 h later the signal increased by 25% and the curves, with or without serum, were overlapping (Rho+LN+FBS versus Rho+LN), and the dose-response dependence was improved.

Regarding ICG/LN (**Figure S4**, **E**) initially, they displayed a dose-dependent increase of the signal, and the serum did not influence the signal, but after 24 h there was a diminution and linearization of the signal, and the curves for ICG /LN and ICG/ LN +FBS overlapped.

Nanoemulsions incorporating rhodamine (Rho/LN) (**Figure S4**, **F**) were only modestly affected by serum, which appears to stabilize the signal at 24 h, especially in the case of higher concentrations.

Before injecting the ICG-labelled, naringenin-loaded nanoemulsions into animals, an in vitro test was performed with the twofold role: (1) to assess the efficiency of ICG-labelled nanoparticles by comparison with a widely used probe, such as Rhodamine and (2) to verify the stability of the signal, since ICG was reported to be susceptible to degradation and subsequent decreased absorption in aqueous solution [1]. To this aim, increasing amounts of nanoemulsions, corresponding to concentrations of the fluorophore in the range 0-60 µM, were mixed with fetal bovine serum (FBS) to a final concentration of 50% in PBS to mimic the in vivo plasma conditions, and the signal was quantified with the aid of the IVIS Spectrum Caliper 200 system. The following filter pairs (wavelengths for excitation/ emission) were used: 745 nm/820 nm for ICG, and 535 nm/620 nm for Rhodamine.


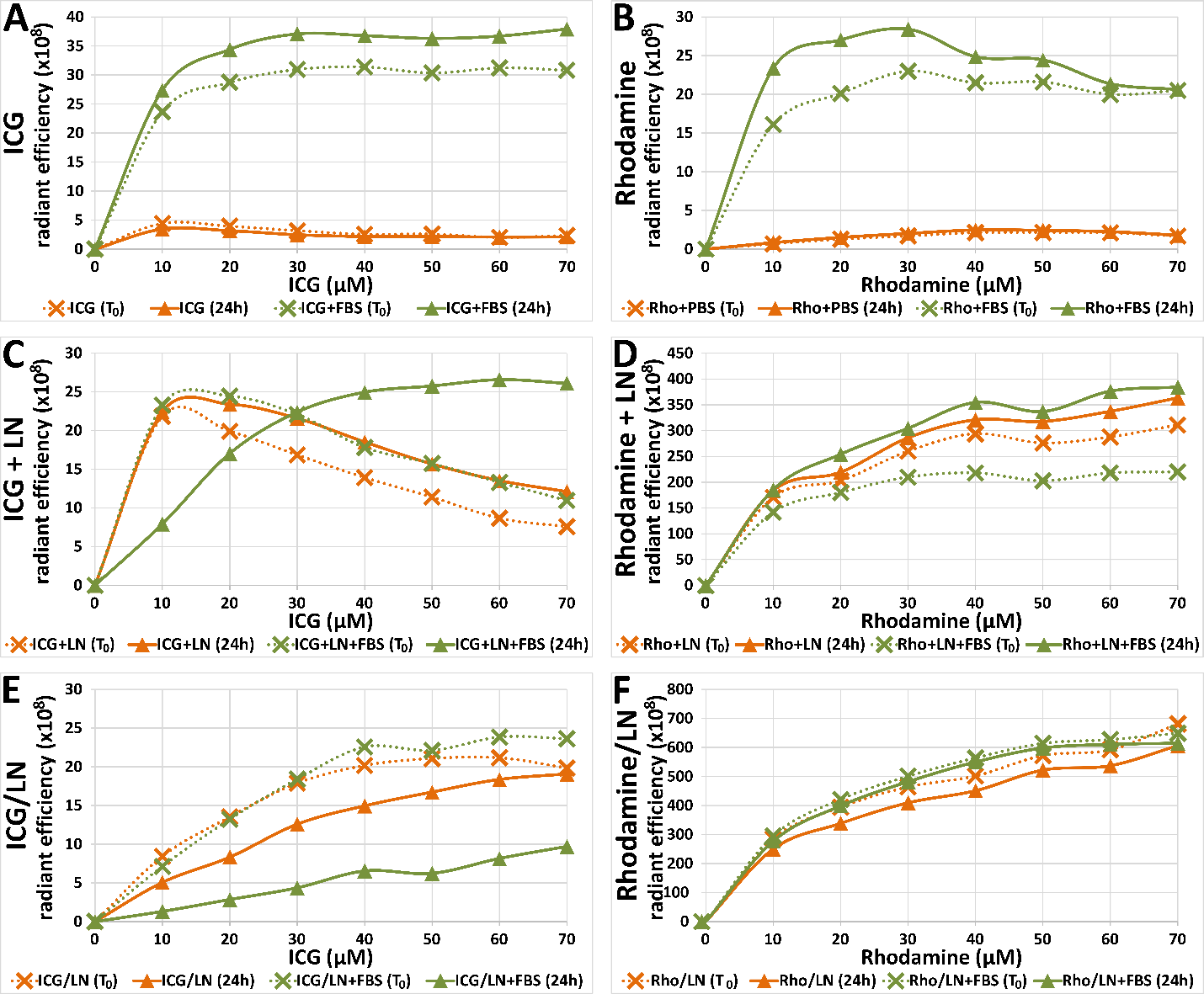


**Figure S4.** Evaluation of radiant efficiency of ICG (**A**, **C**, **E**) as compared with Rhodamine-PE (**B**, **D**, **F**), maintained in the dark for 24 h in the absence or presence of FBS in various microenvironments: fluorophores as free substances in PBS (**A**, **B**), added to pre-formed nanoemulsions (**C**, **D**), or stably incorporated into nanoemulsions (**E**, **F**).

The results indicated that ICG at its emission wavelength (820 nm) was about 10-fold less radiant than Rhodamine at its emission wavelength (620 nm) (**Figures S5A** and **S5C** as compared with **Figures S5B** and **S5D**). The behavior of the two probes after storage for 24 h at room temperature was also different. Thus, the radiant efficiency decreased more pronouncedly for ICG as compared with Rhodamine. Linear regression analysis indicated that R2 varied from 0.8752 to 0.9965 for ICG and from 0.7027 to 0.8678 for Rhodamine. These results indicated a better linearity of the signal for the investigated range of concentrations in the case of ICG, an important criterion to be met for quantitating the accumulation of nanoparticles. Of note, at ICG concentrations higher than 60 µM, the signal started to decrease, probably as a consequence of fluorescence quenching (**Figure S3**).


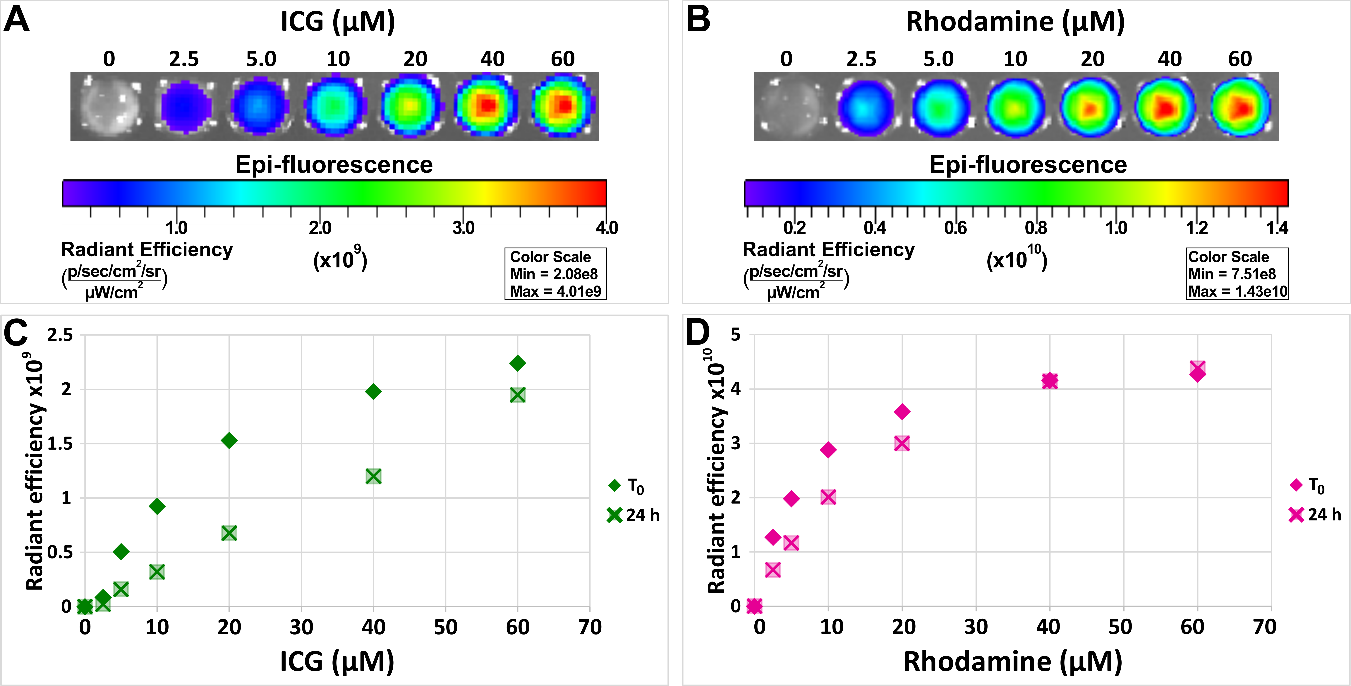


**Figure S5.** Radiant efficiency of ICG (A, C) compared with Rhodamine (B, D) as a fluorescent probe for the naringenin-loaded nanoemulsions. The radiant efficiency of naringenin-loaded nanoemulsions labeled with ICG or Rhodamine-PE incubated in 50% FBS in PBS was measured with an IVIS Spectrum Caliper 200 system. The upper panels (A, B) illustrate the radiant efficiencies of ICG, respectively Rhodamine for the indicated concentrations at the initial time point. The lower panels (C, D) depict graphical representations of the radiant efficiencies of naringenin-loaded nanoemulsions with increasing concentrations of the fluorescent probe at the initial time (T_0_) and after storage for 24 h at room temperature.

Thus, ICG-loaded nanoemulsions displayed a dose-dependent fluorescent signal in the presence of 50% FBS, although it underwent a diminution upon 24 h storage at room temperature, in the dark. It was noticed that the decrease in the signal at 24 h correlated inversely with ICG concentration such that at 60 µM the signal retained ~90% of its initial value. This agrees with the fact that concentrated ICG solutions are more stable [2].

4. Biodistribution of naringenin/ICG-loaded nanoemulsions

The raw values of the radiant efficiencies per g tissue, averaged per experimental groups, are summarized in Table S2.

The control for autofluorescence was represented by the PBS and LPS animals, which did not receive ICG and had similar radiant efficiencies in all organs. However, the autofluorescence level varied significantly across the organs, from as low as 10% in the spleen to as much as 85% in the aorta, whereby percentages were calculated based on the values for the Nar/ICG/LN group.

**Table S2.** Radiant efficiencies (per g tissue), based on ICG fluorescence, of the organs from mice with LPS-induced inflammation, which received non-targeted (Nar/ ICG /LN) or VCAM-1 targeted (V-Nar/ICG/LN) nanoemulsions, at one-hour post-injection. Control (of autofluorescence) is represented by PBS- and LPS-injected animals.

| Radiant efficiency/g tissue | Control  (% from Nar/ICG/LN) | Nar/ICG/LN | V-Nar/ICG/LN |
| --- | --- | --- | --- |
| Plasma (×10^8^) | 1.041 ± 0.049 (27%) | 3.85 ± 0.127 | 3.253 ± 0.198 |
| Spleen (×10^8^) | 0.141 ± 0.009 (10%) | 1.416 ± 0.310 | 1.953 ± 0.424 |
| Aortic root (×10^8^) | 0.302 ± 0.009 (19%) | 1.550 ± 0.270 | 2.400 ± 0.255 |
| Aorta (×10^7^) | 4.985 ± 0.015 (85%) | 5.927 ± 0.698 | 7.860 ± 0.627 |
| Liver (×10^7^) | 0.370 ± 0.025 (13%) | 2.890 ± 0.225 | 2.790 ± 0.235 |
| Heart + aorta (×10^7^) | 0.519 ± 0.007 (19%) | 1.643 ± 0.147 | 2.733 ± 0.230 |
| Kidneys (×10^7^) | 0.450 ± 0.029 (27%) | 1.610 ± 0.150 | 1.700 ± 0.158 |
| Lungs (×10^7^) | 0.797 ± 0.001 (45%) | 1.770 ± 0.045 | 1.907 ± 0.096 |
| Brain (×10^6^) | 3.100 ± 0.300 (75%) | 4.000 ± 0.133 | 4.087 ± 0.419 |

5. Evaluation of PECAM-1 gene expression by real-time PCR

PECAM gene expression was evaluated in the organs harvested from the mice with LPS-induced inflammation that received non-targeted (Nar/LN) or VCAM-1 targeted (V-Nar/LN) naringenin-loaded nanoemulsions (**Figure S6**). Data indicated that there were no significant changes of PECAM expression relative to ACTB in any of the experimental groups. This agrees with data from the literature [3] reporting PECAM-1 as a non-inducible gene upon inflammation.


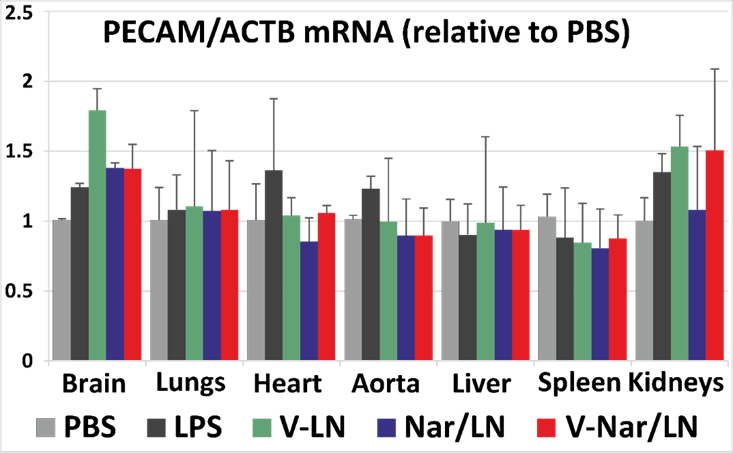


**Figure S6.** Gene expression of PECAM-1 in organs harvested from C57 BL/6 mice that were injected intravenously with non-targeted (Nar/LN) or VCAM-1 targeted (V-Nar/LN) naringenin-loaded nanoemulsions, administered retro-orbitally, in a murine model of LPS-induced inflammation. As controls, mice receiving PBS, LPS or VCAM-1 targeted empty nanoemulsions (V-LN) were used. Results were normalized to ACTB (β-actin) and are expressed as fold changes compared with the PBS-treated mice, considered as 1. The data are expressed as mean ± SD. There were 3 animals per group, except Nar/LN which consisted of 4 animals.

References

1. Gathje, J.; Steuer, R.R.; Nicholes, K.R. Stability studies on indocyanine green dye. *J. Appl. Physiol*. **1970**, *29*, 181–185.
2. Saxena, V.; Sadoqi, M.; Shao, J. Degradation kinetics of indocyanine green in aqueous solution. *J. Pharm. Sci*. **2003**, *92*, 2090–2097.
3. Granger, D.N.; Stokes, K.Y. Differential regulation of leukocyte-endothelial cell adhesion In: *Endothelial cells in health and disease*; Aird, W.C., Ed., Taylor and Francis: Boca Raton, USA **2005**, pp. 229–244.

**Publisher's Note:** MDPI stays neutral with regard to jurisdictional claims in published maps and institutional affiliations.

| 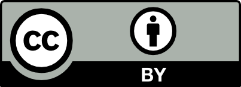 | © 2020 by the authors. Submitted for possible open access publication under the terms and conditions of the Creative Commons Attribution (CC BY) license (http://creativecommons.org/licenses/by/4.0/). |
| --- | --- |
